# Surgical Diagnosis of Malignant Pleural Mesothelioma: 20 Years’ Experience at a High-Volume Referral Center

**DOI:** 10.3390/jcm10091973

**Published:** 2021-05-04

**Authors:** Amedeo Iaffaldano, Thomas Charrier, Filippo Lococo, Diane Damotte, Antonio Bobbio, Marco Alifano, Ludovic Fournel

**Affiliations:** 1 Thoracic Surgery Department, Cochin Hospital, APHP.CUP, Paris University, 75014 Paris, France; amedeog.iaffaldano@gmail.com (A.I.); thomas.charrier@aphp.fr (T.C.); antonio.bobbio@aphp.fr (A.B.); marco.alifano@aphp.fr (M.A.); 2 Università Cattolica del Sacro Cuore, 00168 Rome, Italy; filippo.lococo@policlinicogemelli.it; 3 Thoracic Surgery Department, Policlinico Gemelli, 00168 Roma, Italy; 4 Pathology Department, Cochin Hospital, APHP.CUP, Paris University, 75014 Paris, France; diane.damotte@aphp.fr

**Keywords:** mesothelioma, thoracoscopy, pleurodesis, morbidity, diagnosis

## Abstract

Despite advances, malignant pleural mesothelioma (MPM) remains a challenging disease in terms of diagnosis, treatment, and overall management. Herein, we analyzed, in a large-scale single-center cohort, the characteristics and perioperative course of patients undergoing surgical diagnosis of MPM. We identified a total of 514 consecutive patients, 71.4% male and 28.6% female, with mean age 71.3 +/− 13.6 years. Most exhibited pleural, respiratory, or general symptoms and American Society of Anesthesiologists (ASA) score was ≥3 in 68.3% of cases. Thoracoscopy was the most frequent approach (92.0%) and short open thoracotomy was performed in the remaining patients. Pleurodesis was simultaneously performed in 74.3% of cases. Diagnostic failure led to redo surgery in 3.7% of patients. Non-epithelioid histology was found in 19.5% of MPMs and was significantly more frequent in right-sided MPM (*p* = 0.04), and in patients without history of cancer (*p* = 0.03), or pleural nodules at thoracoscopy (*p* = 0.01). Minor only or major complications occurred in respectively 7.8% and 3.6% of cases. They were more frequent in patients ≥ 70 years (*p* = 0.05) and Performance Status > 2 (*p* = 0.05). The mean hospital stay was 7.5 days. The 30-day and 90-day early mortality rates were 2.3% and 6.4%, respectively. Surgical diagnosis of MPM is a reliable procedure but is associated with significant morbidity and hospital-stay duration.

## 1. Introduction

Despite significant improvements in the management of solid cancers over recent decades, malignant pleural mesothelioma (MPM) remains a challenging disease in terms of diagnosis, treatment, and overall management [1,2]. This malignancy is associated with particularly poor oncological outcome and, considering the natural history of MPM and the avoidance of asbestos exposure, its incidence in industrialized countries is likely to stay stable or even increase and reach a peak in the next few years [3,4]. It seems, thus, mandatory to address this public health issue through different levels of the whole patients’ course. Often delayed, due to an unspecific clinical presentation, one of the key steps in the strategy of care for MPM is the baseline pathological assessment [5]. This diagnosis is often performed surgically, as it allows large pleural biopsies and simultaneous pleurodesis in case of associated effusion. However, this invasive procedure is not trivial, not always uneventful, and frequently requires postoperative in-hospital monitoring [6]. The aim of this study was to evaluate, in a large-scale and single-center cohort, the characteristics and perioperative course of patients undergoing surgical diagnosis of MPM.

## 2. Methods

We retrospectively reviewed all patients who underwent a surgical procedure for diagnosis of pathologically proven MPM, in our department, from 2000 to 2020. Demographic data and clinical characteristics were collected, as well as perioperative macroscopic observations, and adverse-events occurring during the postoperative period. “Major” complications were defined as follows: need for reoperation or unexpected stay in intensive-care unit, acute respiratory distress syndrome, and pulmonary embolism, in contrast to other “minor” complications such as atelectasis, atrial fibrillation, or prolonged air leak. The 8th edition was used for clinical classification of MPMs in patients for whom preoperative computed tomography (CT) imaging was available or staging reported in medical records [7].

As general rule, patients exhibiting a suspected pleural malignancy were referred for surgical diagnosis in our department where a mini-invasive thoracoscopic approach was usually preferred. Accurate pleural cavity exploration, large pleural biopsies (preferentially including the under-pleural adipose tissue) and talc-poudrage pleurodesis were performed, allowing histopathological typing of MPM and pleural symphysis for recurrence prevention, as previously described by our team [8]. In the absence of significant pleural effusion, an elective open approach could be chosen for obtaining malignant pleural tissue. Patients who were not in too poor condition were usually home-discharged at day-1 after chest-tube removal, waiting for permanent pathological diagnosis. In 2010 we began to introduce indwelling pleural catheter in patients exhibiting symptomatic recurrent effusion despite prior talc-poudrage. Descriptive statistics were expressed as mean ± standard deviation for quantitative variables and as frequencies (percentages) for categorical ones. Correlation between factors was assessed using Chi square or Fisher’s exact tests where appropriated. A *p* value < 0.05 was considered as statistically significant. The research was carried out according to principles outlined in the Helsinki declaration and in agreement with French laws on biomedical research and institutional guidelines. Approbation from our institutional review board was obtained for this study (CLEP Decision N°: AAA-2021-08015).

## 3. Results

We identified a total of 514 consecutive patients who underwent a surgical procedure for diagnosis of MPM in our center during the study period. There were 367 (71.4%) men and 147 (28.6%) women, and their mean age was 71.3 +/− 13.6 years. At baseline presentation, most patients exhibited pleural, respiratory, or general symptoms (for details, see Table 1). In 351 (68.3%) patients the preoperative risk evaluation showed an ASA score ≥3.

Pleural biopsies were surgically obtained using thoracoscopic approach in 92.0% of the interventions (*n* = 473), whereas a short open thoracotomy was performed in the remaining patients (*n* = 41, 8.0%), who usually exhibited a thick pachypleuritis without significant pleural effusion. At thoracoscopic evaluation, evident pleural nodules were observed in 67.3% of cases and pleural invasion was suspected in the following locations: mediastinal (31.2%), diaphragmatic (43.3%) visceral (60.0%), and/or parietal (87.2%) pleura, macroscopically. An aspect of inflammatory/hypervascular pleura was observed solely or associated with pachypleuritis in 6.4% and 18.0% of cases, respectively. Chemical pleurodesis was simultaneously performed during thoracoscopic exploration in 74.3% of cases. A pathological diagnosis and assessment of the histological subtype was generally obtained in the first attempt (Table 1), except in 19 patients (3.7%) who required redo-surgery for diagnostic failure or insufficient tumor-tissue sampling. The analysis of pre- and intra-operative factors potentially associated with the histological subtype of MPM revealed that a non-epithelioid histology was significantly more frequent in right-sided MPM (*p* = 0.04), and in patients without history of cancer (*p* = 0.03), or pleural nodules at thoracoscopy (*p* = 0.01). Other clinical and biological characteristics, reported in Table 1, were not statistically correlated to histology.

The early postoperative course was uneventful in 455 (88.6%) patients, whereas minor only or major complications occurred in 41 (7.8%) and 19 (3.6%) cases, respectively. The 30-day and 90-day early mortality rates were 2.3% and 6.4%, respectively. Mean hospital stay was 7.5 ± 5.0 (range 1–27) days. Analyzing risk factors of postoperative complications, we found that only Performance Status (PS) > 2 (*p* = 0.05) and age ≥ 70 years (*p* = 0.05) were significantly associated with higher morbidity after surgery. Also, there was a trend showing that left sided MPM exhibited more complications than right-sided ones (13.5% versus 8.9%, respectively *p* = 0.08).

At univariate analysis, hospital stay was significantly longer in patients exhibiting a PS > 2 (*p* < 0.001), a cT parameter ≥ 3 (*p* = 0.002), white blood cell count > 7 × 10^9^/L (*p* = 0.002), and platelets count > 300 × 10^9^/L (*p* = 0.03). Other characteristics, such as gender, tobacco consumption, asbestos exposure, body mass index, history of cancer, and occurrence of respiratory symptoms, were not statistically correlated to morbidity or postoperative day of hospital discharge.

During the 2010–2020 period, we readmitted a total of 45/344 (13.1%) patients for indwelling pleural catheter insertion.

## 4. Discussion

This large-scale study confirms that the hospital course for baseline surgical diagnosis of MPM is not an anecdotal step of the whole management of affected patients. Indeed, with an associated morbidity rate of 11.4% almost including one third of major complications, this intervention should not be considered as poorly invasive when evaluating the overall benefit/risk balance. Particular caution should be taken in tenuous condition patients, like those presenting with a PS > 2 or a systemic inflammation clinically or at laboratory tests. Nevertheless, the diagnostic efficiency combined with the ability to perform concomitant pleurodesis of surgical procedures offer significant advantage compared to alternative approaches like CT-guided pleural biopsy [9]. Moreover, reliability and rapidity of the histopathologic specimen obtention is mandatory for appropriate prognosis prediction, treatment assignment, and choice of the strategy of care [5,10].

Despite the reported morbidity, surgical biopsy by thoracoscopy or elective mini-thoracotomy accelerates the whole management of MPM by offering sufficient pleural tissue, especially in difficult diagnoses like in desmoplastic subtypes [11], and by allowing in many patients a relatively early attempt of pleurodesis. In our experience, talc poudrage has more probability to succeed when the visceral pleura is not too thickened, thus at the earliest times of most MPM development. Except in the few patients eligible for multimodal treatment including pleurectomy-decortication, in whom it could make the dissection more complex and favor the associated complications [12], first-intent pleural symphysis is probably preferential. Indeed, it avoids repeated pleurocenteses or indwelling catheter insertion both requiring dedicated management and complicating with adverse-events such as chronic pain or device infection [13]. The latter complication is likely to occur relatively early after drain placement, particularly in a context of the patient receiving immunosuppressive systemic treatments like platinum salt-based chemotherapy. Although a randomized study suggests that indwelling pleural catheter is superior to talc pleurodesis for treatment of malignant pleural effusions from different etiologies [14], in terms of total all-cause hospital stay, the overall benefit of the indwelling catheter is likely more relevant in poor condition patients exhibiting metastatic pleural disease than in MPM cases who are at the initial diagnostic phase of their disease [15]. Furthermore, studies comparing palliative interventions such as video-assisted thoracoscopy VATS partial pleurectomy (sometimes associated with indwelling catheter insertion) to talc pleurodesis, concluded in the absence of survival benefit of more invasive treatments while associated with higher morbidity [16,17]. Our department’s policy is generally to propose indwelling catheter insertion secondarily, after baseline clinical work-up, multidisciplinary meeting decision on treatment assignment, and in case of symptomatic recurrence of pleural effusion, altering patients’ quality of life [18,19]. This explains the relatively low rate of readmission for permanent pleural drainage (13.1%) that we report here, although it is probably underestimated, as some patients of the study cohort were likely treated in other institutions than our tertiary referral center.

With respect to the pathological analysis, distribution of the different histologic subtypes of MPM among our 20-year-long consecutive series is similar to those of large epidemiologic studies, showing a very high proportion of the epithelioid subtype [20,21]. It should be noted that the association of non-epitheliod histology with the absence of pleural nodules at intraoperative thoracoscopic evaluation, may leave it prone to the achievement of large pleural biopsies and frozen section analyses, especially in such cases with less typical presentation. Our study was not designed for evaluating the diagnostic sensitivity and specificity of surgical biopsy nor to compare it with less-invasive techniques, evaluated in previous analyses, like trans-bronchial or trans-esophageal biopsies [22,23]. However, the 3.7% rate of redo surgery for diagnostic failure highlights the complexity of pathological assessment and the usefulness of large pleural sampling, especially in the era of molecular and tumor microenvironment analyses [5,24,25]. Moreover, it should be noted that “classical” medical thoracoscopy has also been reported as a relevant and reasonable alternative for pleural biopsy and simultaneous talc poudrage, nevertheless exhibiting lower efficiency than surgical procedures in reaching both diagnostic and therapeutic objectives [26].

## 5. Conclusions

Surgical diagnosis of MPM is a primary step in the whole process of disease management, notably allowing associated pleurodesis in patients who require it, but is associated with significant morbidity and hospital-stay duration. It should always be put in balance when considering alternative and the most recent diagnostic and therapeutic strategies.

## Figures and Tables

**Table 1:** Characteristics of patients.

Variable		*n* (%), Mean +/− SD
Gender	- Female - Male	147 (28.6) 367 (71.4)
Age (years)		71.3 +/− 13.6
BMI (Kg/m^2^)		25.2 +/− 4.4
History of cancer	- Yes - No	116 (22.6) 398 (77.4)
Asbestos exposure (identified)	- Yes - No	236 (46.0) 278 (54.0)
Asthenia	- Yes - No	129 (25.0) 385 (75.0)
Weight loss (last 3 months)	- Yes - No	142 (27.6) 372 (72.4)
Chronic cough	- Yes - No	96 (18.6) 418 (81.4)
Dyspnea	- Yes - No	361 (70.3) 153 (29.7)
Chest pain	- Yes - No	142 (27.6) 372 (72.4)
Smoking	- Yes - No	253 (49.3) 261 (50.7)
Tobacco consumption (pack-years)		28.5 +/− 19.6
Side	- Right - Left - Bilateral	278 (54.1) 197 (38.3) 39 (7.6)
ASA score	- I - II - III - IV	8 (1.6) 155 (30.1) 345 (67.2) 6 (1.1)
PS	- 0 - 1 - 2 - 3 - 4	4 (0.7) 235 (45.7) 185 (36.1) 77 (14.9) 13 (2.6)
Hb (g/dL)		13.1 ± 2.1
WBC (10^9^/L)		6.2 ± 4.5
Plt (10^9^/L)		274 ± 179
cT parameter (*n* = 247)	- 1 - 2 - 3 - 4	100 (40.5) 48 (19.4) 76 (30.8) 23 (9.3)
cN parameter (*n* = 247)	- 0 - 1 - 2 - 3	215 (87.0) 26 (10.5) 6 (2.5) 0 (0.0)
Histological type	- Epithelioid - Sarcomatoid - Biphasic (mixed) - Desmoplastic	414 (80.5) 20 (3.9) 68 (13.2) 12 (2.4)
Re-do surgery	- Yes - No	495 (96.3) 19 (3.7)

BMI: body mass index (kg/m^2^); ASA score: American Society of Anesthesiologists score; PS: performance status; Hb: serum hemoglobin (g/dL); WBC: white blood cell count.

## Data Availability

All generated data are contained within the article. Further information is available on request from corresponding author.
